# E-mental health interventions for men: an urgent call for cohesive Australian policy and investment

**DOI:** 10.1093/heapro/daae033

**Published:** 2024-04-08

**Authors:** Melissa J Opozda, Murray Drummond, Himanshu Gupta, Jasmine Petersen, James A Smith

**Affiliations:** College of Medicine and Public Health, Flinders University, University Dve West, Casuarina, Northern Territory 0810, Australia; Freemasons Centre for Male Health and Wellbeing, South Australian Health and Medical Research Institute and University of Adelaide, North Tce, Adelaide, South Australia 5000, Australia; College of Education, Psychology, and Social Work, Flinders University, Sturt Rd, Bedford Park, South Australia 5042, Australia; SHAPE Research Centre, Flinders University, Sturt Rd, Bedford Park, South Australia 5042, Australia; College of Medicine and Public Health, Flinders University, University Dve West, Casuarina, Northern Territory 0810, Australia; College of Education, Psychology, and Social Work, Flinders University, Sturt Rd, Bedford Park, South Australia 5042, Australia; SHAPE Research Centre, Flinders University, Sturt Rd, Bedford Park, South Australia 5042, Australia; College of Medicine and Public Health, Flinders University, University Dve West, Casuarina, Northern Territory 0810, Australia

**Keywords:** men, e-mental health, mental health, policy, Australia

## Abstract

Few men who experience a common mental disorder access any mental healthcare from a health professional. E-mental health (eMH; online) interventions may facilitate men’s access to mental healthcare and reduce inequities in access via assistance in a format that aligns with their preferences and needs. Recent reviews show that men who have used these programmes generally find them useful and satisfactory; however, data on their effectiveness and factors impacting effectiveness in men’s use are limited. Few eMH interventions have been designed specifically to suit men’s preferences and circumstances, and little is known about the eMH-related experiences and needs of men from vulnerable and marginalized backgrounds. Despite their potential, Australian men’s health, digital mental health, preventive health, and Aboriginal and Torres Strait Islander mental health and social and emotional well-being policies—and men’s health policies from Malaysia, Ireland, and South Africa—make no specific mention to progressing the use or evaluation of eMH for men. We present a series of policy recommendations, aiming to improve men’s access to effective and acceptable mental health assistance via eMH and increase health professional confidence to recommend and support these programmes. These include (i) the need for specific, connected health policy actions and targeted funding; (ii) policy priorities for intervention development, dissemination and evaluation; and (iii) for the experiences of men, particularly those from marginalized and vulnerable backgrounds, to be centred in policy. eMH for men should be promoted alongside interventions to reduce systems and social-level determinants of men’s mental health inequities.

**Contribution to Health Promotion**

- Few Australian men who experience mental ill-health access mental healthcare from a health professional.
- E-mental health (eMH) interventions tailored to men’s preferences and needs may facilitate men’s care access and reduce health inequities.
- Australian health policies, and international men’s health policies, lack specific levels for action on developing and evaluating eMH for men.
- Specific, cohesive policy responses are likely to increase investment in eMH for men, increase men’s access to effective, acceptable mental healthcare, and improve men’s mental health and well-being.
- Integrated health policy responses are required to invest and scale men’s eMH strategies at national, international and global levels.

## THE CHALLENGE OF MEN’S MENTAL HEALTHCARE

As in many countries, men’s mental health remains a considerable challenge in Australia. Nearly one in every five men (18%) fulfil diagnostic criteria for an anxiety, affective (mood) or substance use disorder per year (Australian Bureau of Statistics, 2022), and there has been no improvement in the rate of male deaths by suicide in the last decade (2011: 16.2 per 100 000, 2021: 18.2 per 100 000; Australian Institute of Health and Welfare, 2023b). Furthermore, few men access mental healthcare: in 2021, only 37% of males with an anxiety, affective or substance use disorder accessed assistance for their mental health from a health professional (compared with 55% of females; Australian Bureau of Statistics, 2022). Factors identified as impeding men’s access to care include cost, low availability of appointments outside working hours (Yousaf *et al*., 2015), and men and health professionals not recognizing that behaviours associated with traditional forms of masculinity (e.g. aggression and risk-taking) may be indicators of common mental health issues (Zajac *et al*., 2022). Traditional masculine norms such as stoicism and self-reliance (Seidler *et al*., 2016) and fear, stigma and shame (Wahto and Swift, 2016) can also inhibit men’s help-seeking. Furthermore, men may prefer to seek assistance online or from family or friends (Beyondblue, 2016) or to monitor their symptoms before deciding whether to see a doctor (Smith *et al*., 2008).

Given these barriers and preferences, e-mental health (eMH; also known as online or digital interventions) may align well with men’s needs and wants for mental health assistance. eMH may also work to reduce inequities in healthcare access experienced by marginalized and vulnerable groups (Schueller *et al*., 2019) of men such as culturally and linguistically diverse men (particularly young men of colour), men who are unhoused, men living with a disability, men in rural and remote areas, and sexually and gender diverse men who often experience disproportionately high rates of mental ill-health (Australian Institute of Health and Welfare, 2023a) and are commonly considered ‘hard to reach’. However, though many thousands of free or low-cost mental health apps are accessible online, the vast majority have little or no evidence supporting their effectiveness (Torous *et al*., 2018; Larsen *et al*., 2019), and men’s reportedly low uptake of eMH suggests that many existing programmes do not suit their preferences or needs (Robertson *et al*., 2015).

## THE STATE OF THE EVIDENCE ON eMH FOR MEN

Given the apparent contradictions between high availability and potential, and low evidence and engagement, we undertook two systematic reviews to examine whether eMH interventions work for men and what men think about and want from these programmes. While eMH can take a variety of formats (Pineda *et al*., 2023), we examined web- and app-based, self-directed eMH programmes for depression and anxiety that teach psychological strategies, as these may be particularly valuable for men who are unable, who are not ready or who do not wish to access treatment from a health professional.

We found that though few eMH interventions have been designed to suit men’s specific needs and preferences (Opozda *et al*., 2023b, 2024), men have clear ideas about what they want to see in these programmes and what helps and hinders their use of them. Our meta-synthesis (Opozda *et al*., 2024) of eight studies showed that men’s use of eMH was facilitated by their perceptions that apps and technology are inherently motivating and convenient, supportive and encouragement from important others such as a partner or family, a perception that using eMH helped them to gain some control over their own mental health, and positive mental health and relationship outcomes. Men were less likely to use eMH where they felt that they did not have enough time, believed that they would not benefit from the use and if there were technical issues such as apps not working properly. Men said that they wanted bright, easy-to-use interventions that were relevant to their individual circumstances or that could be easily tailored to them, with content presented in multiple formats (e.g. text and videos), which included psychoeducation, training in psychological strategies, self-monitoring and self-assessments to facilitate the development of helpful skills and behaviours, information on further resources and the ability to access to health professional support if they desired.

Our meta-analysis (Opozda *et al*., 2023b) found considerable current uncertainty in the data related to the effectiveness of eMH interventions for men. We were unable to include 177 papers on mixed sex or gender eMH trials because they had not presented gender or sex disaggregated outcome data, leaving just seven includable papers. In those, eMH use was associated with significantly improved depression symptoms at postintervention (data from four studies), but we found no difference in postintervention outcomes between eMH and control conditions (three studies). The extent of the bias caused by the unavailable data is unclear. Further investigation is needed to understand the effectiveness of eMH interventions for men and the factors influencing their effectiveness. However, men who had used these programmes were largely satisfied with them and found them useful.

We also saw that the literature was derived largely from a narrow population of men predominantly aged in their 30s and 40s from higher-income, Western countries, with little examination to date into the eMH experiences and needs of marginalized and vulnerable groups of men. While eMH interventions appear acceptable (and may have positive effects) for a narrow population of men, it is clear that work is needed to develop and evaluate interventions that have been designed for the specific needs and circumstances of men from a wider range of situations and backgrounds. Men are not one homogenous group, and a range of intersecting aspects of men’s identities and circumstances, such as their age, gender, ethnicity, race, culture, sexual orientation, location and health symptoms and experiences, are all likely to influence their eMH needs and preferences. As such, interventions should be closely co-designed with men from the particular intervention target population in order to increase programme acceptability, engagement and effectiveness (Opozda *et al*., 2023a).

Given the increasing availability of online mental health interventions, their potential to facilitate men’s access to assistance and the current uncertainty about their effectiveness, clear policy positions on eMH for men are needed to drive progress in this field.

## AUSTRALIAN HEALTH POLICY, AND INTERNATIONAL MEN’S HEALTH POLICY, ARE FRAGMENTED

Australia is considered to be a leader in men’s health policy, practice and research (Smith *et al*., 2018) and is one of only seven countries worldwide with a national men’s health policy (White and Tod, 2022). Despite the potential of eMH for facilitating men’s access to effective and acceptable mental health treatment, current policy responses to this topic in Australia are diffused and disconnected. The National Men’s Health Strategy 2020–2030 (Department of Health, 2019) describes the need for ‘male-centred information, programs, and services’ (p. 7) and includes mental health as a priority focus. The National Digital Mental Health Framework 2021 (Department of Health, 2021) prescribes engaging ‘vulnerable cohorts’ in the co-creation, design and delivery of digital mental health services (p. 9), identifying gaps in availability and funding for digital mental health services for specific population groups and investing in ‘new and innovative services, tools and programs’ (p. 10). The National Preventive Health Strategy 2021–2030 (Australian Government Department of Health, 2021) promotes ‘embracing the digital revolution’ (p. 10) and ‘promoting and protecting mental health’ (p. 68). The National Strategic Framework for Aboriginal and Torres Strait Islander Peoples’ Mental Health and Social and Emotional Wellbeing 2017–2023 (Australian Government, 2017) notes the utility of culturally appropriate digital mental health services for Aboriginal and Torres Strait Islander people who are at risk of mental illness or living with mild or moderate mental illness. Approaches involving ‘gender-specific promotion of leadership, social and emotional wellbeing and healing’ are also highlighted (p. 20). Though scattered references are made to men, mental health and digital health across policies, no current Australian health policy makes any specific mention to eMH for men.

While the piece is primarily focused on the Australian context, a pragmatic further examination of the other men’s health policies from around the world suggests that fragmented policy on eMH for men may not be isolated to Australia. Malaysia’s men’s health policy (Ministry of Health Malaysia, 2019) background notes that ‘men’s mental health may require different approaches such as online self-help services…’ (p. 12) and includes a strategy to ‘improve availability and accessibility of health service to men’ (p. 26) but makes no recommendation related to developing or using eMH. Ireland’s policy (Department of Health and Children, 2008; Health Service Executive, 2016) states that background consultation highlighted the Internet as a potentially valuable means of providing targeted health information and support to men, but makes no mention of online interventions in its suite of recommendations related to improving men’s mental health. South Africa’s policy (National Department of Health, 2020) recommends developing online solutions that provide ‘tailored, ongoing support to men and boys’ (p. 31) to increase their access to healthcare, but does not specify which health issues should be supported in these interventions. We were unable to analyse the three other national men’s health policies (Iran and Mongolia, not accessible to the authors; Brazil, not available in English). We acknowledge that other health policies from Malaysia, Ireland and South Africa may mention eMH for men, and also note that every country has differing health priorities, healthcare systems and digital health access that may impact the prioritization of eMH for men. However, as seen in Australian health policies, other countries’ men’s health policies also often recognize the potential utility of eMH (or e-health more generally) for men but do not make specific recommendations to drive the use of these programmes.

## EXPLICIT AND TARGETED HEALTH POLICY STRATEGIES AND FUNDING ARE NEEDED

According to the Australian National Mental Health Commission, ‘e-mental health offers one of the greatest invest-to-save opportunities for government and community in mental health’ (Australian Digital Health Agency, 2017, p. 27). We agree and assert that to deliver on this opportunity, more attention needs to be paid to eMH interventions tailored to the needs and circumstances of men via (a) clear health policy action and (b) increased investment in targeted funding. As such, we make the following recommendations to build better healthy public policy. Our recommendations are made with reference to Australian population groups and policies, but may also be applicable to other countries:

1. eMH for men should be included as an explicit priority in relevant health policies, such as Australian policies on men’s health, digital mental health, preventive health and Aboriginal and Torres Strait Islander health and well-being.
2. Health policies and subsequent resourcing investments should be integrated to ensure that the mental health needs of men are not compromised by insufficient policy cohesion.
3. The lived experiences of men, and particularly those from marginalized and vulnerable backgrounds due to factors such as their ethnicity, disability, age, immigration and socioeconomic status, should drive policy reforms on eMH for men.
4. Policy priorities and funding should target:
  a. Investigating the eMH needs and desires of men from a variety of backgrounds and circumstances, with particular need for nuanced, sensitive investigation into the eMH priorities of Aboriginal and Torres Strait Islander men, culturally and linguistically diverse men and other marginalized and vulnerable populations of men.
  b. The development of eMH interventions that address men’s desires for relevant, easy-to-use assistance, centring men’s voices and experiences throughout the co-development, implementation and evaluation of interventions for different populations of men.
  c. Evaluation of short- and long-term mental health and well-being outcomes of using these interventions for men, to build consumer and health professional confidence in eMH.
  d. Building the capacity of health professionals to better understand and respond to the unique mental healthcare needs of men, with specific reference to the potential role of eMH in care and health professionals’ roles in promoting and supporting this type of assistance.
  e. Developing and promoting the use of eMH initiatives among men, utilizing the expertise of individuals experienced in health-related social marketing.

Implementing these policy recommendations will likely increase men’s confidence in and access to suitable, evidence-based eMH to support their mental health self-management and increase health professional confidence to recommend and support effective, appropriate eMH programmes for men experiencing mental ill-health.

This piece focuses on fragmented Australian health policy responses related to men’s eMH to illustrate the need for greater investment in this area at a national level, particularly in a country already considered to be a leader in men’s health (Smith *et al*., 2018). While our initial investigation suggests that other nations’ policies may also lack specific direction on eMH for men, further international scholarship that considers the circumstances, cultures, health systems and policies of other countries and regions—and the relation of these to men’s potential use of eMH—will be important to facilitate global policy responses in this arena and to promote investment in and scale men’s eMH strategies at both national and international levels.

It is important to recognize that although individual-level interventions such as eMH likely have an important part to play in improving men’s mental healthcare access and outcomes, they are just one strategy in a range of options. A vast range of social determinants influence mental health and well-being (Alegría *et al*., 2018) and individual-level interventions such as eMH programmes generally place the burden of engagement largely on the man experiencing mental ill-health. It is crucial that these individual-level interventions are promoted alongside interventions focused on making positive changes in systems and societal-level determinants of inequities in mental health and care access, such as in education and employment, housing, racism and discrimination, and social norms related to masculinities and mental health (Seidler *et al*., 2016; Alegría *et al*., 2018). Multilevel approaches that work to impact multiple important determinants of inequities in men’s mental health and care access will be necessary to effect sustained positive change.
